# Differential Spatial Distribution of TSPO or Amino Acid PET Signal and MRI Contrast Enhancement in Gliomas

**DOI:** 10.3390/cancers14010053

**Published:** 2021-12-23

**Authors:** Lena Kaiser, Adrien Holzgreve, Stefanie Quach, Michael Ingrisch, Marcus Unterrainer, Franziska J. Dekorsy, Simon Lindner, Viktoria Ruf, Julia Brosch-Lenz, Astrid Delker, Guido Böning, Bogdana Suchorska, Maximilian Niyazi, Christian H. Wetzel, Markus J. Riemenschneider, Sophia Stöcklein, Matthias Brendel, Rainer Rupprecht, Niklas Thon, Louisa von Baumgarten, Jörg-Christian Tonn, Peter Bartenstein, Sibylle Ziegler, Nathalie L. Albert

**Affiliations:** 1 Department of Nuclear Medicine, University Hospital, LMU Munich, 81377 Munich, Germany; Adrien.Holzgreve@med.uni-muenchen.de (A.H.); Marcus.Unterrainer@med.uni-muenchen.de (M.U.); Franziska.Vettermann@med.uni-muenchen.de (F.J.D.); Simon.Lindner@med.uni-muenchen.de (S.L.); Julia.Brosch-Lenz@med.uni-muenchen.de (J.B.-L.); Astrid.Gosewisch@med.uni-muenchen.de (A.D.); Guido.Boening@med.uni-muenchen.de (G.B.); Matthias.Brendel@med.uni-muenchen.de (M.B.); Peter.Bartenstein@med.uni-muenchen.de (P.B.); Sibylle.Ziegler@med.uni-muenchen.de (S.Z.); Nathalie.Albert@med.uni-muenchen.de (N.L.A.); 2 Department of Neurosurgery, University Hospital, LMU Munich, 81377 Munich, Germany; Stefanie.Quach@med.uni-muenchen.de (S.Q.); Niklas.Thon@med.uni-muenchen.de (N.T.); Louisa.vonBaumgarten@med.uni-muenchen.de (L.v.B.); joerg.christian.tonn@med.uni-muenchen.de (J.-C.T.); 3 Department of Radiology, University Hospital, LMU Munich, 81377 Munich, Germany; Michael.Ingrisch@med.uni-muenchen.de (M.I.); sophia.stoecklein@med.uni-muenchen.de (S.S.); 4 Center for Neuropathology and Prion Research, LMU Munich, 81377 Munich, Germany; Viktoria.Ruf@med.uni-muenchen.de (V.R.); Rainer.Rupprecht@medbo.de (R.R.); 5 Department of Neurosurgery, Sana Hospital, 47055 Duisburg, Germany; Bogdana.Suchorska@sana.de; 6 Department of Radiation Oncology, University Hospital, LMU Munich, 81377 Munich, Germany; Maximilian.niyazi@med.uni-muenchen.de; 7 German Cancer Consortium (DKTK), Partner Site Munich, German Cancer Research Center (DKFZ), 69120 Heidelberg, Germany; 8 Department of Psychiatry and Psychotherapy, University of Regensburg, 93053 Regensburg, Germany; christian.wetzel@ukr.de; 9 Department of Neuropathology, University Hospital Regensburg, 93053 Regensburg, Germany; markus.riemenschneider@ukr.de

**Keywords:** TSPO PET, amino acid PET, FET PET, glioma, contrast enhancement, spatial correlation

## Abstract

**Simple Summary:**

Radiotracers targeting the translocator protein (TSPO) have recently gained substantial interest, since TSPO is overexpressed in malignant gliomas, where it correlates inversely with patient’s survival. The high-affinity TSPO PET ligand [^18^F]GE180 was found to depict tumor areas with a remarkably high contrast and has been shown to provide non-invasive information on histological tumor grades. Yet, its significance was questioned with the argument, that the high contrast may solely arise from nonspecific accumulation in tissue supplied by leaky vessels. This study aimed to address this question by providing a detailed evaluation of spatial associations between TSPO and amino acid PET with relative contrast enhancement in T1-weighted MRI. The results show that [^18^F]GE180 contrast does not reflect a disrupted blood–brain barrier (BBB) only and that multi-modal imaging generates complementary information, which may better depict spatial heterogeneity of tumor biology and may be used to individualize the therapy for each patient.

**Abstract:**

In this study, dual PET and contrast enhanced MRI were combined to investigate their correlation per voxel in patients at initial diagnosis with suspected glioblastoma. Correlation with contrast enhancement (CE) as an indicator of BBB leakage was further used to evaluate whether PET signal is likely caused by BBB disruption alone, or rather attributable to specific binding after BBB passage. PET images with [^18^F]GE180 and the amino acid [^18^F]FET were acquired and normalized to healthy background (tumor-to-background ratio, TBR). Contrast enhanced images were normalized voxel by voxel with the pre-contrast T1-weighted MRI to generate relative CE values (rCE). Voxel-wise analysis revealed a high PET signal even within the sub-volumes without detectable CE. No to moderate correlation of rCE with TBR voxel-values and a small overlap as well as a larger distance of the hotspots delineated in rCE and TBR-PET images were detected. In contrast, voxel-wise correlation between both PET modalities was strong for most patients and hotspots showed a moderate overlap and distance. The high PET signal in tumor sub-volumes without CE observed in voxel-wise analysis as well as the discordant hotspots emphasize the specificity of the PET signals and the relevance of combined differential information from dual PET and MRI images.

## 1. Introduction

Gliomas are the most common primary brain tumors and are associated with a dismal prognosis [1]. One of the major challenges is the depiction of the extent of the tumors as well as the intra-tumoral heterogeneity, which is essential for individualized treatment planning. Magnetic resonance imaging (MRI) still represents the diagnostic gold standard for brain tumor imaging. Areas of contrast enhancement (CE), which represent areas of disturbed tight junctions between endothelial cells of the blood–brain barrier (BBB), are usually defined as target volumes for surgical resection and radiotherapy [2]. However, it is increasingly being recognized that additional imaging, e.g., positron emission tomography (PET) using radiolabeled amino acids, is extremely helpful for the assessment of vital tumor tissue beyond CE on MRI images [3,4].

While PET with amino acid tracers has been established as a non-invasive method for glioma imaging depicting an upregulation of amino acid transporters in tumor cells [3,5], the 18-kDa translocator protein (TSPO) has been proposed as novel target for glioma imaging, although being less specific for tumor cells [6]. Up to now, it was assumed that TSPO is not only overexpressed by tumor cells but also by activated microglia or macrophages [7,8], and has therefore been addressed in numerous studies investigating neuro-inflammatory/degenerative diseases [9,10,11,12,13,14]. A recent study with multiple sclerosis patients indicated that increased TSPO signal in the human brain might rather reflect a high cell density of microglial cells and not their activation phenotype [5]. Although it is not yet clarified to which extent inflammatory cells such as tumor-associated microglia/macrophages contribute to the signal of TSPO PET in gliomas, TSPO PET has been suggested to be an interesting tool for glioma characterization, as a positive correlation of TSPO expression with histological tumor grade [15,16] and a negative association with patient’s survival have been reported [17].

Multi-modal imaging using contrast enhanced MRI and dual PET addressing different targets within the tumor and its microenvironment seems therefore a promising approach for a comprehensive spatial tumor characterization. First preclinical and clinical studies using high affinity TSPO PET ligands, e.g., [^18^F]GE180 or [^18^F]DPA-714, have shown that tumor volumes on TSPO and amino acid PET or MRI images differ between modalities [18,19,20,21]. For [^18^F]GE180, however, the suitability of the tracer has been questioned due to the suspicion that the PET signal may represent solely nonspecific “accumulation of the radioligand and its radiometabolites in areas where the BBB is broken” [22,23].

Thus, the goal of this study was to further clarify this issue by using local imaging information from PET signals with information from contrast enhanced MRI on a voxel-by-voxel basis. The investigated multi-modal data for glioma assessment comprised TSPO ([^18^F]GE180, *S-N,N*-diethyl-9-(2-[^18^F]fluoroethyl)-5methoxy-2,3,4,9-tetrahydro-1*H*-carbazole-4-carboxamide) PET, amino acid ([^18^F]FET, *O*-(2-[^18^F]fluoroethyl)-L-tyrosine) PET, and T1-weighted MRI, where voxels from contrast enhanced relative to native T1-weighted MRI images were used as signature of BBB integrity [2]. In order to further quantify the level of the BBB leakage, the ratio comparing pre- and post-contrast-enhanced T1-weighted MRI was assessed as previously described [24]. In a previous study [21], overlap measures of manually defined tumor volumes have been investigated and confirmed the complementary character of the three imaging modalities. The current study extended this approach and used observer independent analyses assessing intra-tumoral heterogeneity by voxel-wise correlation of image information. In addition, spatial overlap as well as distances of tumor hotspots within each modality were investigated.

## 2. Materials and Methods

### 2.1. Patients

In this study, patients at initial diagnosis with a suspected glioblastoma were included consecutively. A [^18^F]GE180 PET scan, a [^18^F]FET PET scan, and MRI scans were acquired within 1 week (mean 7 ± 8 days) and prior to any therapeutic intervention. This was followed by histopathological and molecular genetic classification (e.g., mutation of the *IDH1/2* gene, codeletion of chromosomes 1p and 19q) using tissue samples obtained from stereotactic biopsies or by tumor resection as defined in the revised version of the World Health Organization (WHO) classification for tumors of the central nervous system [25]. Tissue samples were extracted at the Department of Neurosurgery, University Hospital, LMU Munich, and evaluated at the Center forNeuropathology, LMU Munich. Gliomas without any MRI contrast enhancement were excluded from this study. Five of the included patients were part of the previous study [21].

Moreover, genotyping for a polymorphism of the TSPO gene was performed at the Department of Psychiatry of the University Hospital Regensburg as described previously using blood samples [12]. Thus, the patients were categorized as low, medium, or high affinity binders (LAB, MAB, HAB) [26,27,28].

All patients have given written informed consent to the data analysis. The study was approved by the local ethics committee (approval number 17-457).

### 2.2. Imaging

All PET scans were acquired on a Biograph 64 PET/CT scanner (Siemens Healthineers, Erlangen, Germany) at the Department of Nuclear Medicine of the University Hospital, LMU Munich. To minimize motion artefacts during the scan while maintaining patient comfort, patients were carefully positioned and fixed using a head band. For both PET tracers the scan protocol started with a low-dose CT, which was utilized for attenuation correction. Data were acquired in list-mode and reconstructed using an OSEM3D algorithm with 4 iterations, 21 subsets and 5 mm Gaussian post-reconstruction filter (Siemens Healthineers, Erlangen, Germany), including standard corrections for attenuation, random and scattered coincidences, dead time, and decay. The clinically chosen matrix size for reconstruction of 336 × 336 × 109 was halved in axial direction in order to assimilate voxel dimensions in all three spatial directions, resulting in a nearly isotropic voxel size of 2.036 × 2.036 × 2.027 mm^3^ and corresponding matrix size of 168 × 168 × 109.

As previously described [12,29], [^18^F]GE180 was produced with a FASTlab synthesizer and single-use disposable cassettes (GE Healthcare, The Grove Centre Amersham, UK). The specific activity was 2028 ± 1226 GBq/µmol and the number of syntheses was 27. Following an intravenous bolus injection of 185 ± 14 MBq [^18^F]GE180, emission data were acquired 60–80 min p.i. [30] and reconstructed in a single image. This protocol was chosen, since background normalized uptake ratios have shown clinical relevance for differentiation of histologic WHO grades of gliomas [16] and a good correspondence to distribution volume ratios derived from dynamic PET data in healthy tissue and multiple sclerosis lesions [12,31].

On a different day, dynamic [^18^F]FET PET data were acquired after intravenous bolus injection of 185 ± 18 MBq for 40 min p.i. The specific activity was 343 ± 148 GBq/µmol and the number of syntheses was 30. Late 20–40 min p.i. static summation images were used for quantification of [^18^F]FET PET uptake. These late static images have proven to be clinically relevant especially for the delineation of tumor extent and the differentiation of tumor recurrence from radionecrosis and serve as basis for therapy planning and response assessment [3,5].

The pre-therapeutic MRI scans involved an axial T2-weighted sequence along with T1-weighted sequences before and after intravenous injection of contrast agent (0.1 mmol/kg gadobenate dimeglumine, Gd-BOPTA, MultiHance; Bracco Imaging, Milan, Italy) with an automatic injection system. The parameters of both T1-weighted MRI sequences defined within the scan protocol were: voxel size 0.5 × 0.5 × 1.0 mm^3^, echo time 2.98 ms, repetition time 2100.0 ms, flip angle 9 degrees and 256 mm field of view. The scan protocol for T2-weighted MRI was: voxel size 0.8 × 0.8 × 2.0 mm^3^, echo time 103 ms, repetition time 8220.0 ms, flip angle 90 degrees and 250 mm field of view.

### 2.3. Normalization of Images

**Figure 1:**
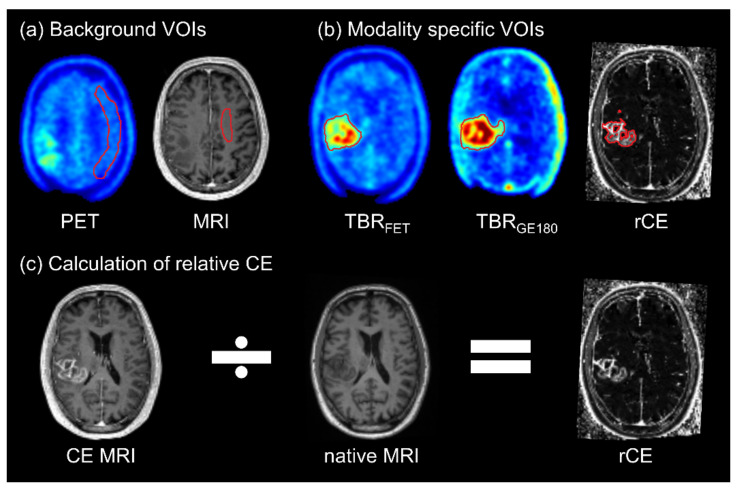
Segmentation and normalization methodology. (**a**) Background VOIs were delineated on the contralesional side. Crescent shaped background volume containing grey and white matter for normalization of static PET images and background VOI in white matter for normalization of T1- and T2-weighted MRI images. (**b**) Exemplary modality specific delineated volumes segmented in tumor-to-background (TBR_FET_, TBR_GE180_), and relative contrast enhancement (rCE) images. (**c**) Derivation of rCE values by voxel-wise normalization of CE MRI data to the respective native MRI values.

[^18^F]GE180 PET and MRI images were registered and resampled to the [^18^F]FET PET image using Pmod Fusion tool (version 4.0, PMOD Technologies, Zurich, Switzerland). For normalization of PET images, a crescent-shaped volume of interest (VOI) was delineated comprising healthy grey and white matter within the contralesional site [32] (Figure 1a). This VOI was used to derive the average background (BG) uptake within each image, which was then utilized for normalization, yielding tumor-to-background ratio images (TBR_GE180_, TBR_FET_).

Images from all three MRI sequences (CE and native T1-weighted MRI, and T2-weighted MRI) were first normalized to healthy white matter intensity defined on the contralesional side (TBR_CE_, TBR_native_, TBR_T2_). The BG-normalized CE T1-weighted MRI image was divided voxel-wise by the corresponding BG-normalized native T1-weighted MRI image, thus creating relative CE (rCE) images (Figure 1c). Since no pre-contrast calibration sequence has been performed, CE MRI intensity normalized to native MRI intensity (i.e., rCE) was assumed to be proportional to the relaxation rate R_1_. Hence, a linear relationship between contrast agent concentration and rCE was assumed, which is valid for sufficiently small concentrations [33,34]. The ratio rCE between pre- and post-contrast measurements was used as surrogate marker for BBB leakage, as applied previously [2,24].

### 2.4. Delineation of Volumes for Voxel-Wise Analyses and Tumor Hotspots

The volume for voxel-wise correlation of rCE, TBR_GE180_, and TBR_FET_ was defined by including all voxels with abnormal signal in at least one modality, also including T2 hyperintense voxels. T2-weighted images were only used to include all suspicious tumor voxels into the voxel-wise analyses. Abnormal signal was defined using threshold-based segmentation within each modality. This was achieved by application of the following iso-contour thresholds. For [^18^F]FET the biological tumor volume was defined using the biopsy proven threshold of 1.6 in TBR_FET_ images [35,36]. Since there is no biopsy-validated threshold for tumor delineation using the other modalities, the remaining thresholds were visually determined by two experienced physicians. The threshold in [^18^F]GE180 images was set to 1.8 in TBR_GE180_ images. This threshold is a consequence of the much lower signal in healthy background for [^18^F]GE180, compared to [^18^F]FET [21,30]. Segmentation of hyperintense tumor volumes in rCE and TBR_T2_ images was performed using a visually defined threshold of 1.3.

Semi-automatic segmentation of volumes using the above-mentioned thresholds was based on an initial manual definition of a confining volume containing all tissue areas suspicious in at least one of the modalities, taking into account a wide safety margin that spreads into healthy tissue, while simultaneously excluding only vessels and healthy ventricles. Within this confining volume, a region growing algorithm was applied for thresholding with an iso-contour starting from user defined seed points. The manual part of the described procedure was performed within the Medical Imaging Interaction Toolkit graphical user interface (MITK; ITK version 4.13.2, VTK version 8.1.0 Qt version 5.12.8 [37,38]) and the automatic region growing was performed with SimpleITK (version 1.2.4, [39]) using Python 3.8.

To assess the distance between hotspots in rCE, TBR_GE180_, and TBR_FET_ images, the hottest 62 voxels (corresponding to the volume of a sphere with diameter of 1 cm) were selected using region-growing, starting from the seed voxel with maximal intensity within each predefined volume. This irregularly shaped region containing the hottest voxels is thus independent of manually placed small spherical volumes.

### 2.5. Statistical Analyses

Correlation plots (TBR_GE180_ vs. rCE, TBR_FET_ vs. rCE, and TBR_GE180_ vs. TBR_FET_) were generated by combining the voxel values obtained from all patients and also on the individual patient level. Pearson’s correlation coefficients and slopes from linear regression were evaluated. Correlation coefficients |r| < 0.3 were considered as none or very weak, 0.3 < |r| < 0.5 as weak, 0.5 < |r| < 0.7 as moderate, and |r| > 0.7 as strong correlation.

The spatial concordance and discordance of hotspot volumes derived from the different images was assessed using Dice similarity coefficients (D) and average Hausdorff distance (AHD). These measures were calculated using the “LabelOverlapMeasuresImageFilter” and the “HausdorffDistanceImageFilter” provided within SimpleITK (version 1.2.4, [39]). The Dice similarity coefficient is calculated using the volumes V1 and V2 from two different modalities and their united volume as [40]

(1) $$D=\frac{2\left|V1\cap V2\right|}{\left|V1\right|+\left|V2\right|}\;.$$

Since the original Hausdorff distance is sensitive to outliers and does only capture the largest direct distance between two volumes, only the symmetric (undirected) average Hausdorff distance was extracted [41,42]:

(2) $$AHD=\max\left(d_{AHD}\left(V1,V2\right),d_{AHD}\left(V2,\;V1\right)\right)\;,$$

where $d_{AHD}\left(V1,V2\right)$ is the directed AHD defined as the average over all distances from points a∈V1 to the respective closest points b∈V2:

(3) $$d_{AHD}\left(V1,V2\right)=\frac{1}{N}\underset{a\in V1}{\sum }\underset{b\in V2}{\min}||a-b||.$$

## 3. Results

### 3.1. Patients

A total of 34 patients at initial diagnosis of a contrast enhancing glioma on T1-weighted MRI images were recruited between 2017 and 2020. A total of 28/34 (82%) patients were diagnosed via stereotactic biopsy and 6/34 (18%) patients via microsurgical tumor resection. In total, 30/34 (88%) gliomas had no mutation of the *IDH1/2* gene and were classified as WHO grade 4. Of the four patients with *IDH1/2* mutation (12%; 3/4 WHO grade 3, 1/4 WHO grade 4), one patient presented with a codeletion of chromosomes 1p and 19q and WHO grade 3. Information on the polymorphism of the TSPO gene was available for 30/34 patients, where 19 high, 7 medium and 4 low affinity binders were identified. For this small patient cohort, differences in standardized uptake values (SUV) of [^18^F]GE180 in the background VOIs between HAB, MAB, and LAB were not significant using 2-sided Kruskal–Wallis test for non-parametric variables (*p* = 0.06, mean SUV ± standard deviation for HAB: 0.34 ± 0.08, MAB: 0.29 ± 0.06 and LAB: 0.26 ± 0.02).

### 3.2. Voxel-Wise Correlation

**Figure 2:**
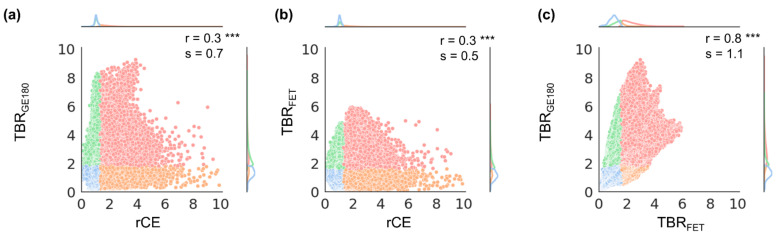
Voxel-wise correlation plots for voxel values from all patients comparing tumor-to-background ratios (TBR) and relative contrast enhancement (rCE): (**a**) TBR_GE180_ vs. rCE, (**b**) TBR_FET_ vs. rCE, and (**c**) TBR_GE180_ vs. TBR_FET_. Color coding of data points illustrates whether the voxel values belong to TBR or rCE positive or negative analysis volumes: Red: Voxels are contained in both analysis volumes, Blue: Voxels are outside both analysis volumes, Orange or green: Voxels are within one analysis volume only. Significances obtained from Pearson’s correlation and the corresponding correlation coefficients (r) and slopes (s) are provided in the upper right corner. Significance was grouped as strong (*p* < 0.001, denoted with ***).

**Figure 3:**
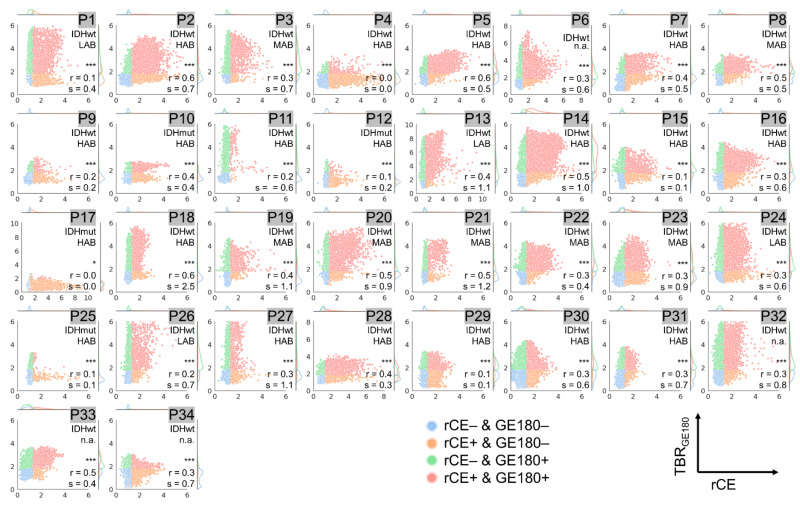
Voxel-wise correlation of [^18^F]GE180 tumor-to-background ratios (TBR_GE180_) vs. relative contrast enhancement (rCE) values for patients P1–P34. The depicted data points are color coded as described in Figure 2: Red: rCE and TBR_GE180_ positive voxels, Blue: rCE and TBR_GE180_ negative voxels, Orange: rCE positive and TBR_GE180_ negative voxels, Green: rCE negative and TBR_GE180_ positive voxels. Significances obtained from Pearson’s correlation and the corresponding correlation coefficients (r) and slopes (s) are provided in the lower right corner. Significance was grouped as strong (*p* < 0.001, denoted with ***), and low (*p* < 0.05, *). The status of *IDH* mutation (IDHmut: with *IDH1/2* mutation; IDHwt: without mutation) and the TSPO-polymorphism genotype are given for each patient in the upper right corner (if not available denoted with n.a.). The axis ranges are all fixed to values between zero and six except for patients P6, P13, P17, and P28 where the upper limit was adapted due to higher voxel values.

**Figure 4:**
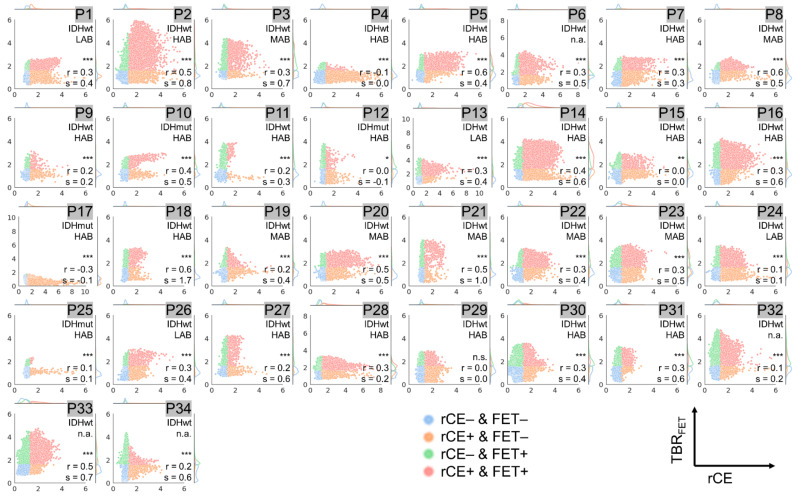
Voxel-wise correlation of [^18^F]FET tumor-to-background ratios (TBR_FET_) vs. relative contrast enhancement (rCE) values for patients P1–P34. The depicted data points are color coded as described in Figure 2: Red: rCE and TBR_FET_ positive voxels, Blue: rCE and TBR_FET_ negative voxels, Orange: rCE positive and TBR_FET_ negative voxels, Green: rCE negative and TBR_FET_ positive voxels. Significances obtained from Pearson’s correlation and the corresponding correlation coefficients (r) and slopes (s) are provided in the lower right corner. Significance was grouped as strong (*p* < 0.001, denoted with ***), medium (*p* < 0.01, **), low (*p* < 0.05, *), and not significant (n.s.). The status of *IDH* mutation (IDHmut: with *IDH1/2* mutation; IDHwt: without mutation) and the TSPO-polymorphism genotype are given for each patient in the upper right corner (if not available denoted with n.a.). The axis ranges are all fixed to values between zero and six except for patients P6, P13, P17, and P28 where the upper limit was adapted due to higher voxel values.

**Figure 5:**
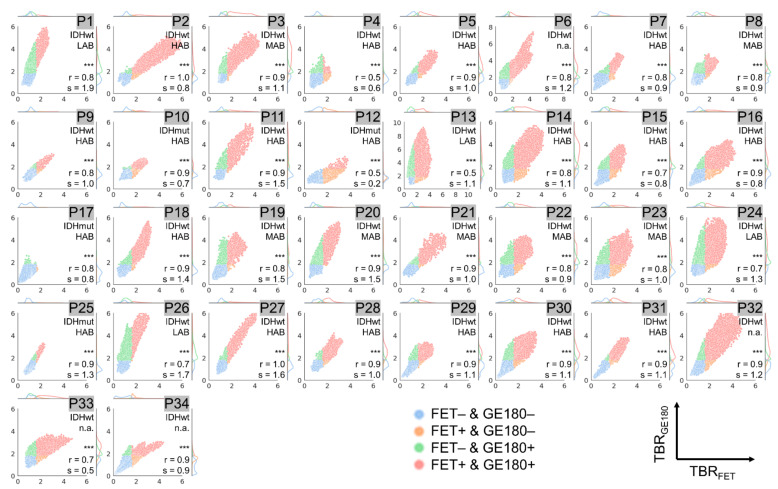
Voxel-wise correlation of tumor-to-background ratios TBR_GE180_ and TBR_FET_ for patients P1–P34. The depicted data points are color coded as described in Figure 2: Red: TBR_FET_ and TBR_GE180_ positive voxels, Blue: TBR_FET_ and TBR_GE180_ negative voxels, Orange: TBR_FET_ positive and TBR_GE180_ negative voxels, Green: TBR_FET_ negative and TBR_GE180_ positive voxels. Significances obtained from Pearson’s correlation and the corresponding correlation coefficients (r) and slopes (s) are provided in the lower right corner. Significance was grouped as strong (*p* < 0.001, denoted with ***). The status of *IDH* mutation (IDHmut: with *IDH1/2* mutation; IDHwt: without mutation) and the TSPO-polymorphism genotype are given for each patient in the upper right corner (if not available denoted with n.a.). The axis ranges are all fixed to values between zero and six except for patients P6 and P13 where the upper limit was adapted due to higher voxel values.

Results from voxel-wise analysis of all included patients combined are presented in Figure 2 and for each individual patient in Figure 3, Figure 4 and Figure 5. The respective overall and individual correlation coefficients from Pearson’s correlation (r) and linear regression slopes (s) are provided within each scatter plot, as well as the status of *IDH* mutation and the TSPO-polymorphism genotype. Taking all data together, there was only weak correlation of rCE and PET (Figure 2a,b), but strong correlation between the PET signals (Figure 2c). This was also found in patient-individual correlations: In the majority of patients, correlations of the two PET signals were strong with several cases with moderate or weak correlation (Figure 5), whereas for rCE and PET an overall weak correlation was found, with no correlation in 50% of all cases (Figure 3 and Figure 4). In most patients, a large proportion of voxels without increased rCE (73 ± 17%) could be identified, of which a high fraction was positive in [^18^F]GE180 PET (46 ± 27%) and [^18^F]FET PET (32 ± 18%) with a wide range of TBR values in both PET modalities (green data points in Figure 3 and Figure 4). The voxels with elevated rCE exhibited also a large variance observable for both PET modalities (red and orange in Figure 3 and Figure 4).

In a subset of patients (P4, P12, P13, P15, and P33), only a weak or moderate correlation between TBR_GE180_ and TBR_FET_ values was detected. Moreover, a large spread of data points not exactly following the regression line for several patients and a broad range of slope and intercept values was observed. For instance, while among strongly correlating values (r > 0.7) similar TBR values with slopes between 0.7 and 1.3 were found in the majority of gliomas (*n* = 21), eight gliomas presented with large slopes above 1.3 (P1, P11, P18, P19, P20, P25, P26, P27). Smaller slopes below 0.7 were only found for tumors with moderate correlation (P4, P12, P33). A high slope indicates a higher contrast in [^18^F]GE180 compared to [^18^F]FET PET. Within this comparison, an intercept of above zero is associated with areas with increased [^18^F]GE180 uptake and no [^18^F]FET signal and below zero the other way round.

### 3.3. Dice Coefficients and Hausdorff Distances for Comparison of Tumor Hotspots

While voxel-wise analyses allow for an assessment of the correspondence of semi-quantitative values for the entire distribution, it was of further interest to evaluate the sub-volume with the most intense signal defined in each separate image to assess tumor heterogeneity. The respective discordant and concordant sub-volume fractions, Dice coefficients and Hausdorff distance measures are provided in Table 1. Average overlap measures of hotspots within TBR and rCE images were low and the corresponding distances were large (rCE vs. TBR_GE180_; rCE vs. TBR_FET_: D 11%; 10%, AHD 12 mm; 14 mm). No overlap of rCE hotspots with TBR_GE180_ or TBR_FET_ hotspots was found in 18/34 (53%) and 22/34 (65%) patients, respectively. Overlap and distance measures of hotspots within both PET modalities implied higher concordance, but still revealed a divergence of the hottest sub-volumes (D 23%, AHD 9 mm), where 12/34 (35%) presented with no overlap of PET hotspots.

**Figure 6:**
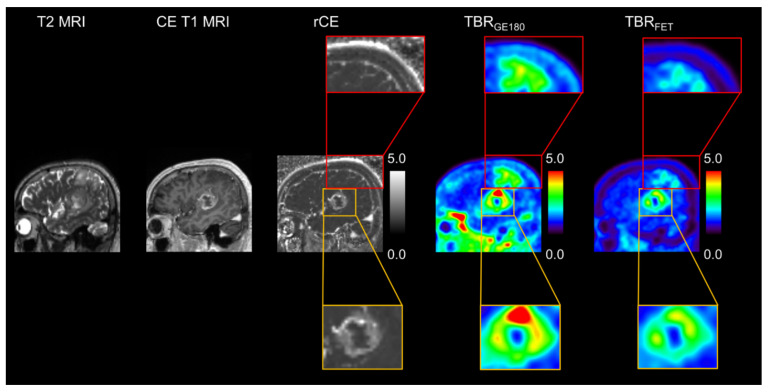
Shown are multi-modal images of one example patient in sagittal view. Depicted are T2-weighted (T2 MRI) and contrast enhanced T1-weighted MRI images (CE T1 MRI) and the generated relative contrast enhancement (rCE) and tumor-to-background ratio (TBR_GE180_, and TBR_FET_) images. Color-bars and intensity ranges are provided for quantitative images. The patient presented with clear tumor specific signal within each modality. In the superior part an additional tumor area without MRI contrast enhancement can be identified using PET, with [^18^F]GE180 PET signal revealing the highest contrast to background.

For visualization one example of a patient with two tumor foci is shown (Figure 6), of which one part shows tumor specific signal in all modalities and the other part presents with increased PET signal in both modalities and no MRI contrast enhancement. Moreover, this second focus exhibits differing TBR levels with markedly increased TBR_GE180_ compared to TBR_FET_.

## 4. Discussion

In this study, [^18^F]GE180 PET, [^18^F]FET PET, and T1-weighted MRI data were correlated voxel-by-voxel to assess regional differences in PET signals within the tumor and to evaluate whether PET signal intensity is correlated with relative contrast enhancement on MRI images. This is of particular interest for TSPO PET imaging using [^18^F]GE180, as the latter has previously been claimed to represent nonspecific tracer accumulation in areas of disrupted BBB [22,23] and a systematic analysis of this issue is still lacking [22,23,43,44,45,46,47,48,49].

For the chosen patient cohort with newly diagnosed suspected high-grade gliomas showing regions with contrast enhancement on T1-weighted MRI images, voxel-wise correlation plots demonstrated no to only moderate correlation of TBR and rCE values. Furthermore, even voxels without signs of increased rCE, in which the BBB can be assumed to be largely intact, presented with high uptake intensity for both radiotracers, [^18^F]GE180 and [^18^F]FET. Additionally, analyses of hotspots using Dice coefficients and average Hausdorff distances revealed no or only very small overlaps and large distances between volumes delineated in TBR_GE180_ and rCE images. Notably, in 53% of patients no overlap of TBR_GE180_ hotspots and rCE hotspots was found. Thus, the high tumor-to-background ratio in [^18^F]GE180 images is clearly not dominated by BBB breakdown and therefore not only due to “the accumulation of the radioligand and its radiometabolites in areas where the BBB is broken”, as previously assumed [22,23]. Likewise for [^18^F]FET, for which the static signal is commonly regarded to have low BBB dependency [50,51], voxel-wise analysis showed low correlation between PET signal intensity and rCE as well as highly diverging hotspots. Hence, the high discrepancy between TBR_GE180_ and rCE supports the conclusion that contrast is primarily driven by specific uptake, while a nonspecific component cannot be excluded. Measuring the specific-to-nonspecific ratio in glioma tissue requires future blocking experiments complementing results from a previous study assessing healthy appearing tissue of multiple sclerosis patients [44]. Since the level of TSPO expression and TSPO ligand binding is exceedingly higher in glioma tissue compared to healthy appearing tissue of multiple sclerosis patients, the specific-to-nonspecific ratio in gliomas is expected to be even higher. Additionally, [^18^F]GE180 has the advantage that the radiometabolites fraction, which is suspected to play a major role for nonspecific uptake, is much lower than for other second generation TSPO ligands [31,52]. Additionally, radiometabolites were reported to be polar and thus less likely to pass the BBB.

For TBR_GE180_ and TBR_FET_ a high agreement of three-dimensional uptake information was observed using voxel-wise correlation in the majority of the high-grade glioma patients evaluated in this study, however, with some remarkable exceptions. Individual regression slopes and intercepts were found to vary considerably and substantial discrepancies in local hotspots were detected, potentially yielding clinically useful complementary information. In detail, while the individual regression slopes of some patients implied an equal increase in TBR values between the two PET modalities, some gliomas presented with a significantly higher and few with a lower increase in TBR_GE180_ compared to TBR_FET_ values, implying a generally higher contrast in [^18^F]GE180 images. It would be interesting to correlate these findings with patient survival and therapy outcome. Such a biologic significance would relate to the previous observation [21] that a high ratio of [^18^F]GE180 to [^18^F]FET is correlated with high tumor aggressiveness. Therefore, the signal intensity ratio may be a promising imaging biomarker for non-invasive prognostication and, by voxel-wise analysis, could be relevant for the identification of the most aggressive tumor sub-volume. This is supported by a case series of three glioblastoma patients [20] reporting that an increased or discordant TSPO signal compared to [^18^F]FET PET predicted areas of tumor progression. Beside these encouraging pilot data, the clinical significance of differing spatial hotspots as prognostic markers is currently not established. In this respect, longitudinal follow-up studies will be of great importance. Since only a weak or moderate correlation between TBR_GE180_ and TBR_FET_ values was found in 5/34 patients, the outcome of these particular patients will be investigated.

One limitation of the study is that voxel-wise correlation analyses can be sensitive to potential image registration errors, which, however, are relatively low in the case of rigid brain registration. Another limitation is that relative contrast enhancement is not a direct measure of BBB permeability. In addition to a non-linear saturation of rCE values with increasing concentrations of contrast agent, the contrast agent itself allows only for the visualization of severe BBB disruption. Rather subtle BBB changes without severe breakdown of tight junctions between endothelial cells may not be visible. However, although relative contrast enhancement comparing pre- and post-contrast enhanced T1-weighted MRI data may not perfectly reflect the level of BBB disruption, it is acknowledged to be the best clinically used indicator of BBB disturbance [2,24]. Although we found high tracer signals in areas without MRI contrast enhancement, we cannot exclude that a low degree of BBB impairment, which may have been clinically invisible on MRI images, might still be required to enable a sufficient tracer availability at the target tissue site, at least for [^18^F]GE180. We therefore recognize that a negative [^18^F]GE180 PET signal in areas without contrast enhancement should be interpreted with caution, as it may be caused by either lack of TSPO expression or completely intact BBB. However, the high amount of voxels without contrast enhancement but with increased [^18^F]GE180 signal as well as the high level of tracer intensity in these voxels clearly show that [^18^F]GE180 PET provides complementary information to MRI, which may be of value for the clinical management of glioma patients.

A further potential confounding factor is the blood signal fraction within tumors and its contribution to MRI and PET signals. From previous studies, blood signal contributions to the applied static PET images can be estimated to be approximately 20% for [^18^F]GE180 and 10% for [^18^F]FET in both, healthy and tumor tissue [21,31,53]. Despite the low influence, the exact contributions of the blood volume fraction to the PET and MRI signals will need to be evaluated using dynamic PET and MRI studies. The inclusion of perfusion weighted imaging and pharmacokinetic modelling might be helpful to evaluate the influence of general microvasculature changes within tumor tissue compared to healthy tissue, such as blood volume fraction, blood flow, and BBB permeability.

Taken together, we could show that TSPO PET, amino acid PET and T1-weighted MRI provide differential information and are therefore interesting complementary imaging tools for glioma characterization. Further systematic analyses of the PET radiotracer uptake processes and related confounding factors, as well as a spatial correlation with stereotactic biopsies are needed to evaluate in more detail the potential clinical benefit of combined TSPO PET, amino acid PET, and MRI information. In this context, it will be essential to assess the relevance of the different hotspots detectable within the various modalities, and parametric maps taking into account dynamic PET and MRI information could provide further important insights, which have proven relevance for, e.g., glioma classification [54].

## 5. Conclusions

Spatial distributions of TBR values in TSPO ([^18^F]GE180) and amino acid ([^18^F]FET) PET images, and relative contrast enhancement on T1-weighted MRI images show individual signal patterns in gliomas. The lack of correlation of MRI signal with PET signals as well as the highly diverging hotspots suggest that the signal of both, [^18^F]GE180 and [^18^F]FET, is not dominated by BBB breakdown. The voxel-wise multi-parametric mapping as proposed in the current study may in the future allow for the three-dimensional characterization of the individual tumor and the accurate depiction of tumor heterogeneity. This should facilitate individualized therapy planning and provide prognostic information.

## Figures and Tables

**Table 1:** Presented are values averaged over all patients and the corresponding standard deviations (mean ± SD) of discordant and concordant sub-volume fractions, Dice coefficients, and average Hausdorff distance measures of hotspots defined in tumor-to-background ratio (TBR) and relative contrast enhancement (rCE) images. The descriptions of the compared volumes V1 vs. V2 are provided in the column labels. Sub-volumes are given as the percentage of the combined volume V1 ∪ V2. V1\V2 corresponds to the sub-volume where V1 exceeds V2, and V2\V1 is the sub-volume where V2 exceeds V1. V1 ∩ V2 is the overlapping volume fraction.

Overlap/Distance Measures	rCE vs. TBR_GE180_	rCE vs. TBR_FET_	TBR_FET_ vs. TBR_GE180_
V1\V2	47 ± 6%	47 ± 7%	42 ± 10%
V2\V1	47 ± 6%	47 ± 7%	42 ± 10%
V1 ∩ V2	7 ± 11%	7 ± 13%	16 ± 20%
Dice coefficient	11 ± 17%	10 ± 19%	23 ± 26%
Average Hausdorff distance	12 ± 13 mm	14 ± 12 mm	9 ± 10 mm

## Data Availability

The data presented in this study are available on request from the corresponding author. The data are not publicly available due to ethical restrictions.
